# Frühe urologische und venerourologische Quellen zur Sexualmedizin aus Wien: Protagonisten im Grenzgebiet zweier neuer medizinischer Spezialfächer

**DOI:** 10.1007/s00120-024-02392-6

**Published:** 2024-08-27

**Authors:** Friedrich H. Moll, Heiner Fangerau

**Affiliations:** 1 Institut für Geschichte, Theorie und Ethik der Medizin, Medizinische Fakultät, Centre for Health and Society, Heinrich-Heine-Universität Düsseldorf, Düsseldorf, Deutschland; 2 Museum, Bibliothek und Archiv zur Geschichte der Urologie, Deutsche Gesellschaft für Urologie e. V., Düsseldorf, Berlin, Deutschland; 3 c/o Urologischer Arbeitsplatz Krankenhaus Merheim, Urologische Klinik, Kliniken der Stadt Köln gGmbH, Neufelder Straße 32, 51067 Köln, Deutschland

**Keywords:** Sexualmedizin, Urologie, Wissenschaftsentwicklung, Wien, Fin de siecle, Sexual Medicine, Urology, Development of sience and knowledge in Vienna

## Abstract

Im Jahre 2014 wurde eine „Österreichische Gesellschaft zur Förderung der Sexualmedizin und der Sexuellen Gesundheit“ (ÖGFSSG) gegründet. Diese Gründung blickt zurück auf die spätestens seit der Mitte des 19. Jahrhunderts zunehmenden Bemühungen, das Wissensfeld akademisch zu erschließen. Die Wiener Medizin spielt hier eine eigene, besondere Rolle. Der Beitrag wendet sich zentralen Wiener Akteuren zu, die um 1900 ein besonderes Interesse für die Sexualmedizin aus urologischer Perspektive aufbrachten. Sie arbeiteten im Grenzbereich mehrerer nach Spezialisierung strebender Disziplinen im Umfeld einer rasch wachsenden Großstadt mit multiplen kulturellen Einflüssen. Die hier im Sinne einer Quellensammlung vorgestellten Personen trugen durch ihre Arbeiten zum Aufschwung der Sexualmedizin bei, indem sie sich auf ein Gebiet vorwagten, in dem bis dahin keine medizinische oder andere Disziplin eine Deutungshoheit beanspruchen konnte.

## Abstract (en)

The Austrian Society for the Promotion of Sexual Medicine and Sexual Health (*Österreichische Gesellschaft zur Förderung der Sexualmedizin und der Sexuellen Gesundheit* [ÖGFSSG]) was founded in 2014. This foundation looked back upon the increasing efforts to develop this field of academic knowledge since the middle of the 19th century, in which Viennese medicine played an important role. This article highlights key Viennese players who had a particular interest in sexual medicine from a urological perspective around 1900. They worked in the wider area of several disciplines, striving for specialization in the environment of a rapidly growing metropolis with multiple cultural influences. The scholars presented here as a collection of sources contributed to the upswing in sexual medicine through their work by venturing into an area in which no medical or other discipline had previously been able to claim sovereignty of interpretation.

## Einleitung

**Abb. 1:**
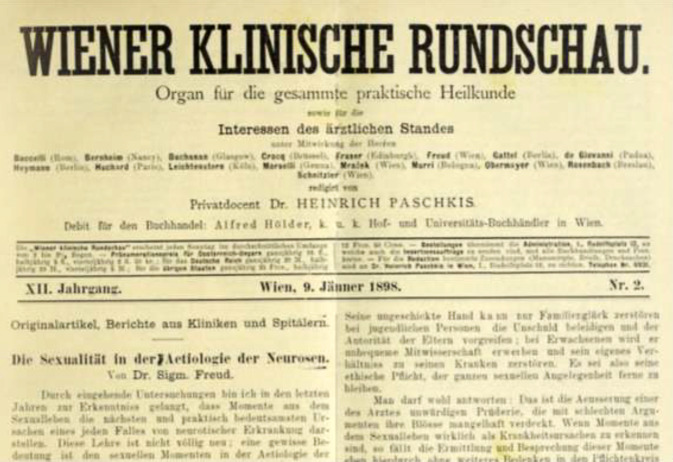
Publikation Sigmund Freuds von 1898 in der vielgelesenen Klinischen Rundschau, die über mehrere Ausgaben verteilt war

Wien gehörte neben Berlin zu den Orten, an denen sich relativ früh Sexualwissenschaft und -medizin entwickeln konnten. Über allem steht hier wie ein Fixstern *Sigmund Freud (1856–1939)* mit seiner Schrift über „Die Sexualität in der Ätiologie der Neurosen“ von 1898, in der er beiläufig auch den Begriff „Sexualwissenschaft“ nutzte^1^ ([1]; Abb. 1).

Auch der 1892 nach Wien berufene Leiter der psychiatrischen Universitätsklinik am Allgemeinen Krankenhaus *Richard von Krafft-Ebing (1840–1902)* gehört unbestritten zu den frühen Pionieren einer sich entwickelnden Sexualwissenschaft und -medizin im Wien des „fin de siècle“. In seinem mehrfach aufgelegten Hauptwerk „Psychopathologia sexualis“ beschrieb und systematisierte er anhand von Fallbeispielen Sexualitäten [2–7]. *Eugen Steinachs (1861–1944)* Hormonforschungen zuletzt werden bis heute als Basis einer geschlechtsspezifischen Hormonforschung und der Entwicklung einer oralen Antikonzeption international erinnert^2^ [8–12].

Relativ unbeachtet blieben indes bisher frühe Schriften von der Urologie nahe stehenden Wissenschaftlern aus dem Kaiserreich Österreich-Ungarn [13]. Dies mag einen Grund darin haben, dass die Geschichte der Sexualwissenschaft und -medizin sich meistens mit einem diskurs- und ideengeschichtlichen Schwerpunkt [14, 15] auf die Felder der Homosexualität [16, 17], der Masturbation [18], die Prostitution [19] oder das Wirken besonders zentraler Akteure konzentrierte. Das klinisch-praktische Handeln der Mediziner, die sich aus Perspektive sehr unterschiedlicher Fachgebiete (von der Inneren Medizin über die Venerodermatologie und die Neurologie/Psychiatrie bis hin zur Urologie) mit der Sexualität und ihren medizinischen Implikationen befassten, fand hier selten Beachtung.

In der Regel jedoch suchten und suchen männliche Patienten mit Therapiewunsch neben ihren Hausärzten des Vertrauens zumeist Urologen bzw. Venerourologen und ggf. früher Badeärzte (Bad Ischl, Karlsbad, tsch. Karlovy Vary, Marienbad, tsch. Mariánské Lázně) oder Neurologen/Psychiater auf [20].

**Abb. 2:**
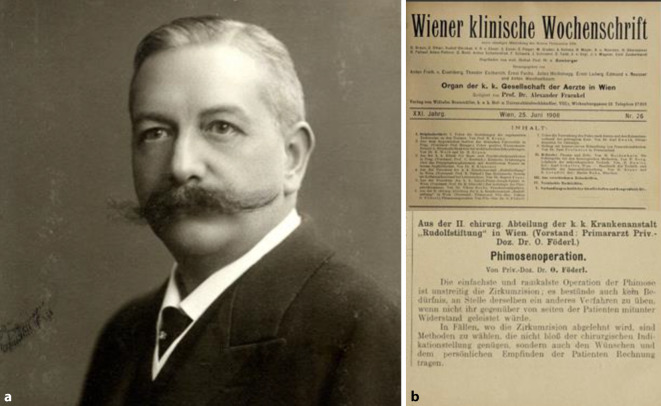
**a** Oskar Foederl (1865–1932), Wienbibliothek im Rathaus, Tagblattarchiv (Fotosammlung, TF-002788) sowie (**b**) Ausriss der Originalarbeit „Phimosenoperation“ aus der renommierten *Wiener Klinischen Wochenschrift*

Ein Beispiel für ein medizinisches Handlungsfeld am Rande zur Sexualmedizin bietet die operative Behandlung der Phimose. Für Wien spielte hier etwa *Oskar Foederl 1865–1932*, Primarius an der k. k.-Rudolfstiftung, sicherlich eine wichtige Rolle. Er stellte in der *Wiener Klinischen Wochenschrift* 1908 eine richtungsweisende Operationstechnik mit Vorhauterhalt vor, die noch heute angewandt wird ([21]; Abb. 2).

Gerade in eher anonymen Großstädten wie Wien fanden Spezialisten auf dem Gebiet der Behandlung des Urogenitaltraktes und der Sexualsphäre ein Umfeld vor, in dem sie von vielen Patienten auch aus einem großen Umkreis aufgesucht wurden, die unter Geschlechtskrankheiten oder deren Folgen oder anderen Funktionsstörungen der Sexualsphäre wie der „männlichen Impotenz“, der „männlichen Sterilität“, der „Neurasthenie“ mit Auswirkungen auf die Potenz oder „Priapismus“ litten. Metropolen wie Wien wurden am Ende des 19. Jahrhunderts als Teil oder sogar Akteur eines Diskurses über Sexualität und als pathologisch begriffene Sexualität erlebt. Zwischen Großstadtleben, Sexualität, Körperlichkeit und Unterhaltung (von Varieté, Theater bis Hygieneausstellungen [22, 23] und Panoptikum) bestand eine enge konzeptuelle Verbindung [24].

Die Diskurse und Frontlinien in der sich neu entwickelnden Sexualmedizin entspannten sich hier im Grenzbereich mehrerer, sich im 19. Jahrhundert etablierenden medizinischen Spezialfächer sowie der Rechtswissenschaften [25] und auch der Philosophie [26, 27]. Diese Grenzbereiche zeichnen sich dadurch aus, dass hier wechselseitige Überschneidungen und Bezugnahmen erfolgten, die das Bild der jeweiligen Fachdisziplinen akzentuierten und bereichern sollten und gleichzeitig das Feld der Sexualmedizin als Grenzobjekt akzentuierten [28]. Christa Putz hat darauf hingewiesen, dass die Schwierigkeit und die Aufgabe darin bestand, einem neuen Tätigkeits- und Wissenschaftszweig eine innere Ordnung und äußere Grenzen gegen benachbarte Gebiete zu geben – und das mit Referenz auf durch Beobachtung abgesichertes, systematisches Vorgehen sowie moralische Neutralität [29].

In Fortsetzung einer Analyse zu Berlin [30] soll in diesem Beitrag entlang der Beiträge zentraler Wiener Akteure (siehe z. B. Figdor [31]) das Arbeiten an den Grenzen der beiden Fachgebiete nachgezeichnet werden. Ausgewählte klinische Themenschwerpunkte und Publikationen mit Bezug zur Sexualwissenschaft werden in biografische Skizzen eingebettet im Sinne einer Quellensammlung vorgestellt, um ein Bild des medizinischen Feldes am Rande zur Sexualwissenschaft zu gewinnen, wie es sich im Wien des „fin de siècle“ bis in die 1920er-Jahre repräsentierte.

## Bezugspunkt Wien

Die Untersuchungsfolie Wien eignet sich hier besonders, da in Wien eine sehr frühe Fachdifferenzierung innerhalb der Medizin stattfand, die sich aber nicht in einer Gründung einer eigenständigen nationalen Fachgesellschaft – Urologie oder Sexualmedizin – widerspiegeln sollte. Gleichzeitig war Wien im ersten Drittel des 20. Jahrhunderts ein wichtiger Ort für die Sexualmedizin und die Urologie:

Unlängst haben sich zwei größere viel beachtete Ausstellungen mit der Sexualwissenschaft in Wien befasst: 2022 gab es im jüdischen Museum Wien die Ausstellung „Love me Kosher“ [32], 2016–2017 zeigte das Wien Museum „Sex in Wien: Lust. Kontrolle. Ungehorsam“ [33]. Hier stellten die Ausstellungsmacher fest, dass Wien sozusagen der „ground zero“ (Bunzl) für die Erfindung der modernen Sexualität(en) war [34].

Bereits beim 1. Deutschen Urologenkongress, der 1907 in Wien stattfand, war ein Vortrag dem „Thema“ „Impotenz“ gewidmet [35, 36]. Der statistisch begründeten Relativierung, [37] bei mehr als 90 Redebeiträgen sei nur einer diesem Themenbereich gewidmet gewesen, kann entgegnet werden, dass die Front- und Verteidigungslinie des jungen Faches Urologie zu dieser Zeit an der Grenze des operativen Faches Chirurgie lag. Dieses kämpfte auf finanzieller und hochschulpolitischer Ebene bis in die 1970 Jahre um die Deutungshoheit über die Urologie^3^. Daher musste ein erster Kongress einer neuen Fachgesellschaft, die sowohl einen operativen wie endoskopischen minimal-invasiven Schwerpunkt vertrat, sich gerade operativ mit den beiden Chirurgischen Universitätskliniken in Wien besonders auseinandersetzen und im Kongressprogramm abbilden, da der Präsident Anton Ritter von Frisch (1849–1917) gerade nicht Ordinarius an der Universität war, sondern Leiter der urologischen Abteilung der Allgemeinen Wiener Poliklinik.

Ende des Jahres 1918/Anfang 1919 war eine „Wiener Urologische Gesellschaft“ etabliert worden (lokale Gesellschaft in Berlin bereits 1911), in der immer wieder Themen im Grenzbereich Sexualmedizin wie auch in der k. k. Gesellschaft der Ärzte erörtert wurden^4^ [38–40].

Im Jahre 1930 fand in Wien ein *Kongress der *„*Weltliga für Sexualreform*“ (WLSR) (1. Kongress 1921 Berlin, Kopenhagen 1928, London 1929, Brünn 1932) statt. Mit über 2000 Teilnehmenden war dieser IV. Wiener Kongress der Weltliga der mit Abstand größte. Das Programm umfasste mehr als 70 Vorträge und Berichte aus der Beratungspraxis sowie einen Ausstellungsteil [41–44].

**Abb. 3:**
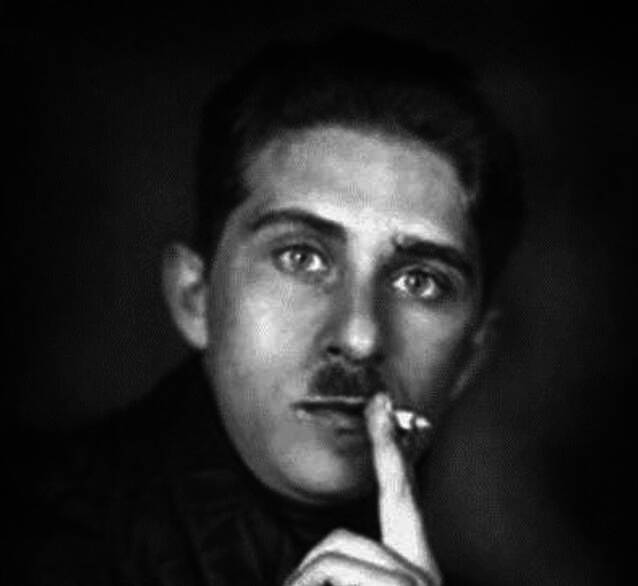
Leo Schidrowitz 1894–1956 (Foto: Privat Genwiki online: https://www.geni.com/people/Leo-Schidrowitz/6000000116744961850 ebenfalls in der Ausstellung *Love me Kosher*, Ausstellung Wien: https://www.jmw.at/ausstellung/love_me_kosher)

Zwei Jahre zuvor hatte 1928 der Wiener Verleger Leo Schidrowitz (1894–1956, 1938 Flucht und Exil nach Paris und Brasilien bis 1949; [45, 46]) in Verbindung mit dem Verlag für Kulturforschung [47] ein Institut für Sexualforschung gegründet. Dieses Institut verstand sich als Spiegelbild des Institutes von Magnus Hirschfeld in Berlin „in anderem Rahmen“ [48]. Schidrowitz hatte Kontakte zu vielen Sexualforschern, allerdings nicht zu Vertretern der Psychoanalyse mit der Ausnahme von Wilhelm Stekel (1868–1940; [49]; Abb. 3).

Das urologisch-andrologisch-sexualmedizinische Grenzgebiet vertraten in dieser Einrichtung Wissenschaftler wie der Leipziger Urologe Hermann Rohleder (1866–1934, [50]), der Berliner Internist Paul Führbringer (1849–1930; [30]) oder der Wiener Oskar F. Scheuer [51]. Leo Schidrowitz war in Wien eine unbestrittene Größe [52]. Der österreichische Schriftsteller Heimito von Doderer (1896–1966) zeichnete von ihm im Roman „Die Dämonen“ ein wenig schmeichelhaftes, antisemitisch gefärbtes Porträt [53]. Das Institut fungierte de facto durch die Personalunion Verleger und Institutsleitung u. a. als Herausgeber verschiedener Buchpublikationen, die an herausragender Stelle wie dem Simplicissimus besprochen wurden [54–56] und übernahm u. a. das „Protektorat“ über den deutsch-schweizer Aufklärungsfilm „Feinde im Blut“ 1931 (Regie Walter Ruttmann). Die Erotik in der Photographie, die geschichtliche Entwicklung der Aktphotographie sowie des erotischen Lichtbildes und seine Beziehungen zur Psychopathia sexualis von Erich Wulffen (1862–1936) gehörte ebenfalls zum Verlagsportfolio [57, 58]. Das Verlagsgeschäft wurde 1935 eingestellt, in den Jahren 1936 und 1937 kam es in Österreich zu mehreren Gerichtsverhandlungen und dem Verbot der Druckerzeugnisse des Verlags wegen Pornographie [52].

Wie Barbara Herzog [59] konstatiert, lässt sich vergleichbaren Verboten zum Trotz auch im Nationalsozialismus eine sexualfreundliche Haltung ablesen, solange die Sexualwissenschaft auf Fortpflanzung und Rasseforschung ausgerichtet war. Ein Beispiel für Wien bietet etwa die 1943 durch die „Wiener Akademie für Ärztliche Fortbildung“ durchgeführte Veranstaltung „Der Mann“, in der etwa das Parteimitglied (NSDAP-Mitgliedsnummer 6.201.678) Koloman Haslinger (1889–1944)^5^ über „Potenzstörungen“ [60] referierte.

**Abb. 4:**
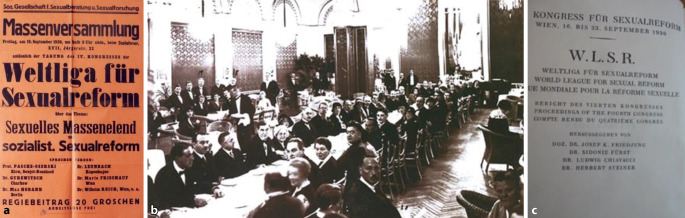
**a** ÖNB (Bildarchiv und Grafiksammlung Signatur: PLA16316209). **b** Sitzungssaal in Wien, Magnus Hirschfeld Gesellschaft (mit freundl. Genehmigung). **c** Verhandlungsband Frontispiz (Repro Moll-Keyn, Sammlung Moll, mit freundl. Genehmigung)

Erst seit 1979 existiert in Österreich eine „Österreichische Gesellschaft für Sexualforschung“ (Ernst Bornemann 1915–1995). In Berlin war schon 1913 eine „Ärztliche Gesellschaft für Sexualwissenschaft und Eugenik“ (AeGeSe; Eulenburg-Hirschfeld-Bloch) gegründet worden (bis 1933) sowie die „Internationale Gesellschaft für Sexualforschung“ (InGeSe; Moll-Marcuse; bis 1932; Abb. 4, 5 und 6).

**Abb. 5:**
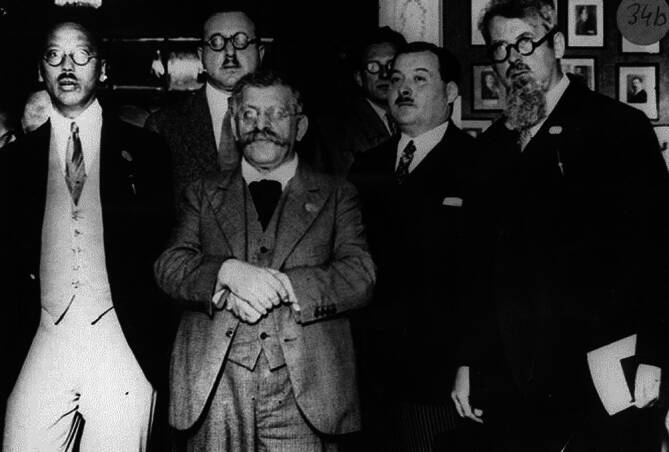
WLSR Kongress Wien 1930: Haire, Norman, 1892–1952; Friedjung, Josef K., 1871–1946; Vachet, Pierre, 1892 – Hirschfeld, Magnus, 1868–1935. (ÖNB Bild und Graphiksammlung NB 523061‑B, mit freundl. Genehmigung)

**Abb. 6:**
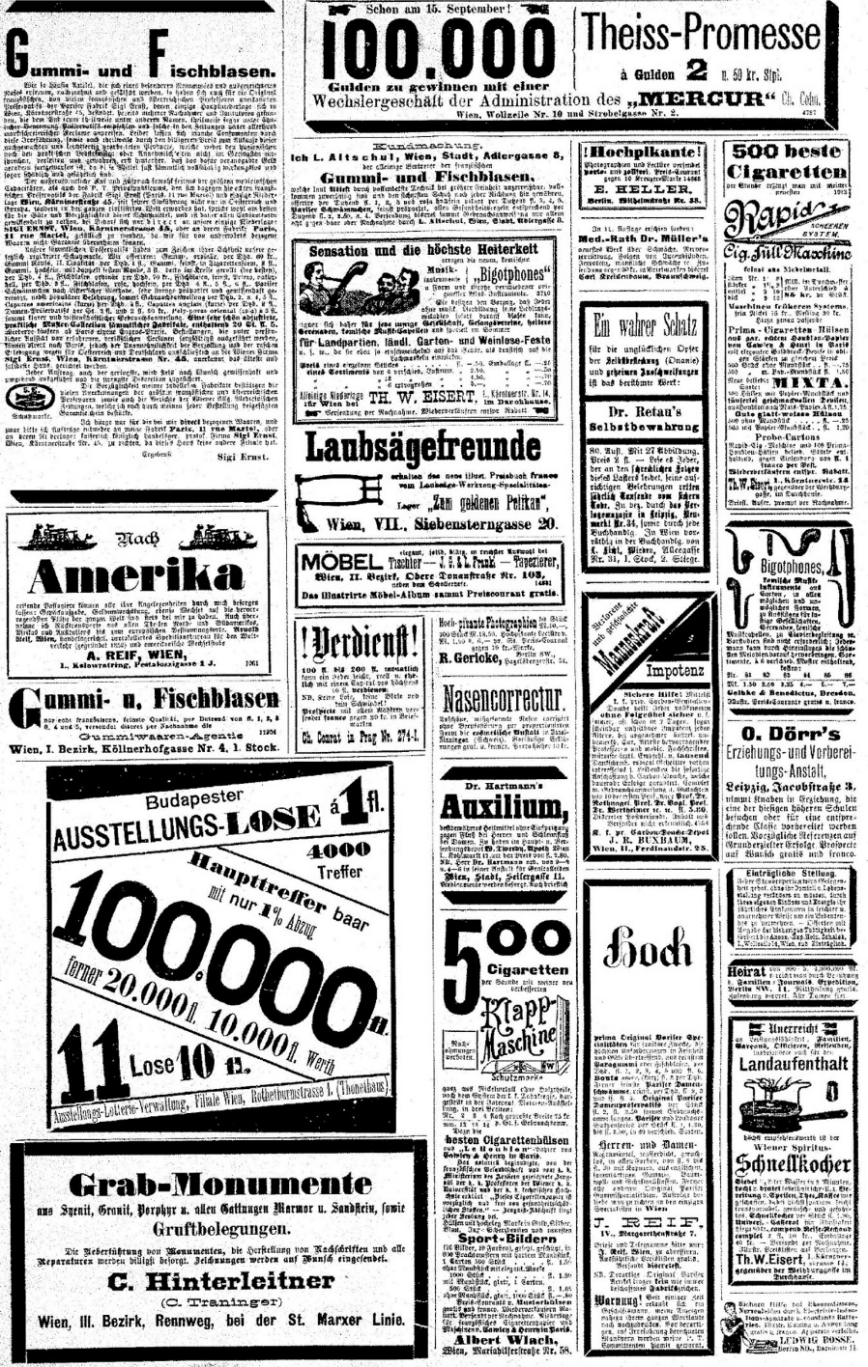
Der Floh, 13.09.1885, 17. Jahrg., Nr. 35. Allein auf einer Seite im Anzeigenteil sind 8 Anzeigen, die für die Zeitgenossen eindeutig der Sexualsphäre zuzuordnen waren, abgedruckt. Dies veranschaulicht den Umgang mit Fragen der Sexualität in der Großstadt Wien an der Wende zum 20. Jahrhundert

## Bioergografische Skizzen

Wir wenden uns nun einigen frühen Akteuren im Grenzbereich zwischen Urologie und Sexualwissenschaft und ihren Beiträgen zum Feld zu in Reihenfolge ihrer Geburt.

### Karl Langer Ritter von Edenberg (1819–1887)

**Abb. 7:**
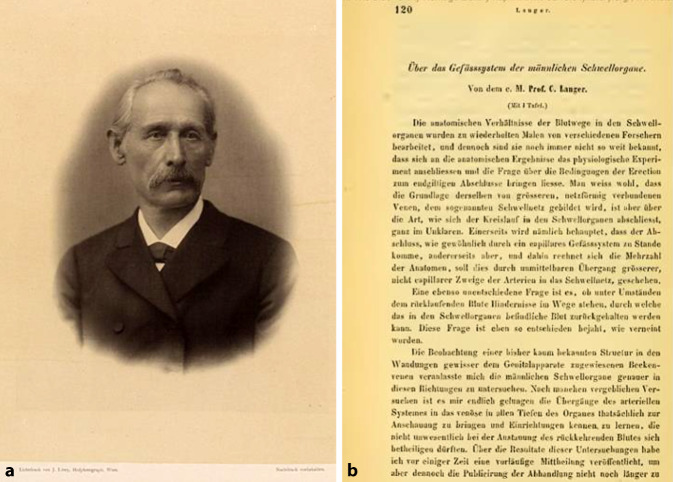
**a** Karl Langer Ritter von Edenberg (1819–1887). (J Löwy, Universität Wien, Signatur: 135.671, mit freundl. Genehmigung). **b** Ausriss Verhandlungsbericht Akademie (Repro Moll-Keyn, mit freundl. Genehmigung)

Karl Langer Ritter von Edenberg war von 1845 bis 1847 als Prosektor unter Joseph Hyrtl (1810–1894) tätig. Ab 1856 lehrte er im Josephinum das Fach „deskriptive Anatomie“ und ab 1874 im I. Anatomischen Institut. In der XIX. Sitzung vom 10. Juli 1862 der „Akademie der Wissenschaften“ [61] hatte er sich grundlegend mit dem Gefäßsystem der Schwellkörper des Penis beschäftigt und erstmalig eine anatomische Beschreibung präsentiert, die später in den Verhandlungsberichten publiziert wurde (Abb. 7).

### Josef Englisch (1835–1915)

Auch Josef Englisch zählt zu den frühen Vertretern einer sich differenzierenden Urologie in Wien. Er schloss sein Medizinstudium im Jahre 1863 in Wien mit dem Erwerb des akademischen Grades eines Dr. med. ab. Danach war er als „Operationszögling“ an der 1. Chirurgischen Lehrkanzel bei Johann von Dumreicher (1815–1880) bis 1865 tätig. 1866 wechselte er bis 1869 als 1. Sekundararzt an die dritte chirurgische Klinik von Leopold von Dittel (1815–1898) im Allgemeinen Krankenhaus (AKH), wo er seine urologische Prägung erhielt.

**Abb. 8:**
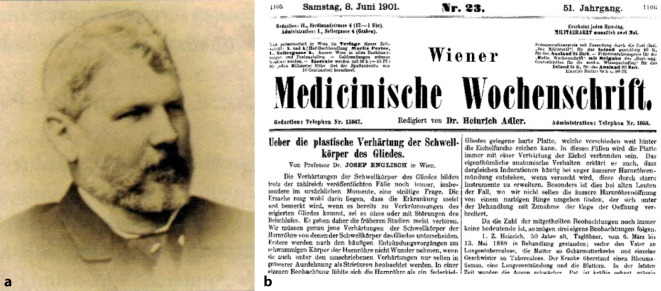
**a** Joseph Englisch 1835–1915. (Bildersammlung DGU, Repro Moll-Keyn, mit freundl. Genehmigung). **b** Ausriss „Über die plastische Verhärtung der Schwellkörper des Gliedes“ (Wien. med. Wochenschrift 1901. Nr. 23, 24 und 25)

Josef Englisch wurde 1876 zum „Primarius“ (Chefarzt) der Chirurgischen Abteilung am Wiener Rudolf-Spital bestellt. Er habilitierte sich als „Chirurg“ 1871 und war ab 1893 a.o. Professor an der Medizinischen Fakultät. Der Forschungsschwerpunkt des im Jahre 1907 als Ehrenmitglied in die Deutsche Gesellschaft für Urologie [31, 62, 63] aufgenommenen Josef Englisch lag auf dem Gebiet der Urologie. Im Grenzgebiet zur Sexualmedizin verfasste auch er 1902 in der *Wiener Medizinischen Wochenschrift* einen Beitrag zur Induratio penis plastica [64, 65]. Für die fachprägende *Real-Encyclopädie* von Eulenburg [66] zeichnete Josef Englisch für die klinischen Lemmata „Beschneidung“, „Penis“, „Hypospadie“ „Präputium“ und „Varicocele“ verantwortlich^6^ (Abb. 8).

### Robert Ultzmann (1842–1889)

Robert Ultzmann gehörte in Wien zu den frühen Protagonisten im Grenzbereich zwischen Urologie und Sexualmedizin. Nach der Habilitation mit einem urologischen Thema im Jahre 1872 wirkte er an der auch von ihm mitgegründeten „Wiener Allgemeinen Poliklinik“, die Zeitgenossen als „erste deutsche Urologische Abteilung für Krankheiten der Harnorgane“ apostrophierten [67]. 1888 wurde Robert Ultzmann zum Mitglied der Leopoldina gewählt [68]. Zunächst verfasste er wissenschaftliche Artikel in der „Wiener medizinischen Presse“ zu Samenerguss, Samenfluss [69] und Sterilität [70] sowie in der „Wiener Klinik“ zu „Neuropathien des Geschlechtstrakts“ [71] und zur „Impotenz“ [72].

**Abb. 9:**
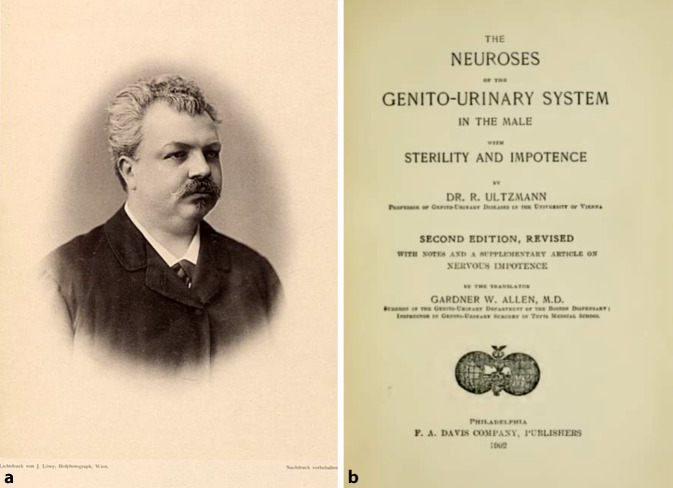
**a** Robert Ultzmann 18842–1889. (Lichtdruck nach J. Löwy, Universität Wien Signatur:135.734; auch ÖNB – Porträtsammlung, Repro Moll-Keyn, mit freundl. Genehmigung). **b** Frontispiz der 2. englischsprachigen US Ausgabe, die 13 Jahre nach seinem Tode erschien. Die Übersetzungen in die englische Sprache verdeutlichen die „Strahlkraft“ (Lesky) der Wiener Medizin und Urologie zu dieser Zeit (Repro Moll-Keyn, mit freundl. Genehmigung)

Für Albert Eulenburgs (1840–1917) „Real-Encyclopädie der gesamten Heilkunde“ verfasste er das Lemma „Impotenz“ [73]. Seine Arbeiten wurden in mehrere Sprachen übersetzt und er galt als ausgewiesener Experte in diesem Gebiet ([74, 75]; Abb. 9).

### Maximilian Edler von Zeissl (1853–1929)

Der Syphidologe Maximilian Edler von Zeissl (1853–1929), Sohn des Dermatologen Hermann von Zeissl (1817–1884), war nach dem Medizinstudium in Wien ab 1880 zunächst als „Operationszögling“ an der I. Chirurgischen Klinik der Wiener Universität im Allgemeinen Krankenhaus unter Johann Heinrich Freiherr Dumreicher von Österreicher (1815–1880) tätig, bevor er 1882 1. Assistent bei dessen Nachfolger Eduard Albert (1841–1900) wurde. Im Jahre 1883 habilitierte er sich während seiner Tätigkeit als Sekundararzt an der Hautklinik seines Vaters zwischen 1882 bis 1884 für „Hautkrankheiten und Syphilis“ [76–78]. 1898 erhielt er den Titel eines k.k.a.o. Professors [79].

**Abb. 10:**
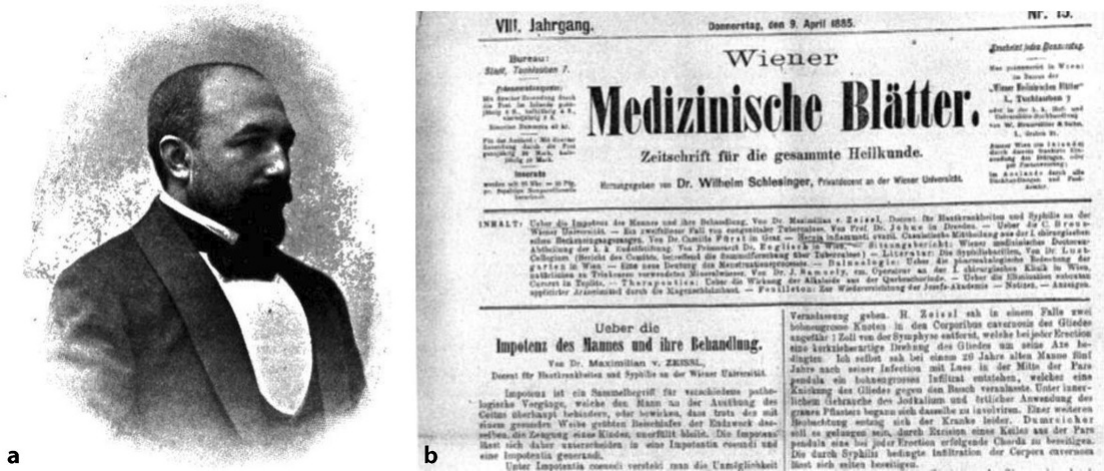
**a** Maximilian Edler von Zeissl (1853–1925). (aus Pagel 1901, Repro Moll-Keyn, mit freundl. Genehmigung). **b** Ausriss „Impotenz des Mannes und ihre Behandlung“ (Repro Moll-Keyn, mit freundl. Genehmigung)

Zum Grenzgebiet zwischen Urologie und Sexualwissenschaften trug er mit dem Artikel „Über die Impotenz des Mannes und ihre Behandlung“ [80] in den *Wiener Medizinischen Blättern* 1885 sowie im Jahre 1890 mit dem „Beitrag zur Anatomie der Lymphgefäße der männlichen Geschlechtsorgane“ [81] wesentlich bei. Im „Handbuch der Harn- und Sexualorgane“ von 1894 war er von Wilhelm Zuelzer (1834–1893) und Felix Martin Oberländer (1851–1915) für das Kapitel „Die acuten Krankheiten der männlichen Harnröhre“ ausgewählt worden ([82]; Abb. 10).

### Ernest Finger (1856–1939)

**Abb. 11:**
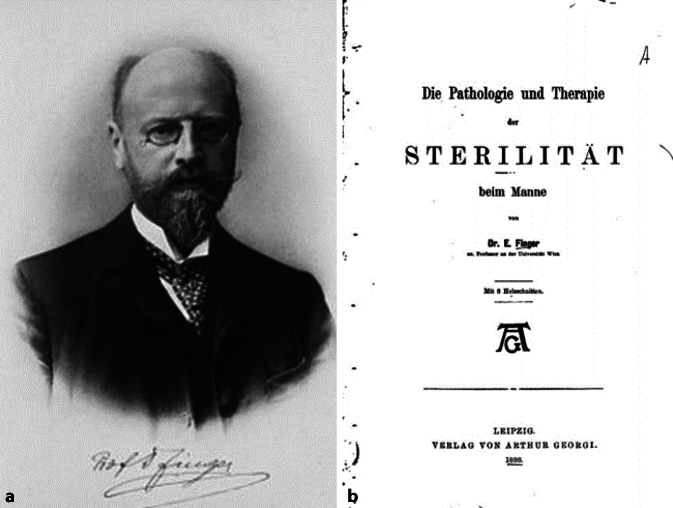
**a** Ernest Finger 1856–1939. (Loewy, Wien um 1904, wikicommons). **b** Frontispiz „Die Pathologie und Therapie der Sterilität beim Mann“ (Repro Moll-Keyn, mit freundl. Genehmigung)

Ernest Finger war besonders auf dem Gebiet der Gonorrhoe und Syphilis aktiv [83, 84]. Er war Vorstand der II. Wiener Universitätsklinik für Haut- und Geschlechtskrankheiten, Präsident der Österreichischen Ärztekammer und Vorsitzender des obersten Sanitätsrates zwischen 1925–1931. Bereits 1888 hatte er ein vielfach aufgelegtes Lehrbuch, „Die Blennorrhöe der Sexualorgane und ihre Complicationen: Nach dem neuesten wissenschaftlichen Standpunkte und zahlreichen eigenen Studien und Untersuchungen dargestellt“^7^, herausgegeben, dem eine umfassende Studie zur männlichen Sterilität im Jahre 1898 folgte [85]. In dieser Publikation handelte er die männliche Impotenz in allen Facetten ab und fügte ein gut recherchiertes Literaturverzeichnis an, das den Wissensstand der Zeit dokumentieren sollte. 1901 demonstrierte er einen klinischen „Fall zur Induatio penis plastica“ vor der Dermatologischen Gesellschaft in Wien ([86]; Abb. 11).

### Robert Lichtenstern (1874–1955)

**Abb. 12:**
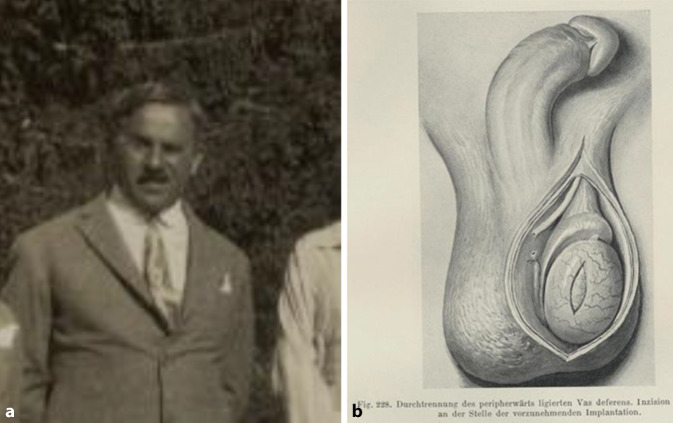
**a** Robert Lichtenstern (1874–1955). (Österr. Nationalbibliothek, mit freundl. Genehmigung). **b** Frontispiz Urologische Operationslehre. Mit 231 z. T. mehrfarbigen Abbildungen im Text. (Berlin und Wien: Urban & Schwarzenberg 1935, S. 271)

Robert Lichtenstern machte sich kurz nach dem Ersten Weltkrieg in Zusammenarbeit mit Magnus Hirschfeld (1868–1935) und Eugen Steinach im Zusammenhang mit Hodentransplantationen zur Verjüngung einen Namen [87, 88]. Zusammen mit Steinach hatte er seit 1916 Kastrations- und Transplantationsversuche am Menschen durchgeführt, um die innersekretorische Funktionswirkung der „Pubertätsdrüse“ mit Auswirkung auf Körperbau und Verhalten nachzuweisen [89–101]. Er war der Überzeugung, dass die Organverpflanzung deutlich wirksamer sei als nur die Übertragung oder Aufnahme eines Organextrakts. Zusammen mit Hirschfeld wollte er gleichzeitig prüfen, ob dieses Verfahren auch zur „Heilung“ der Homosexualität dienen könnte (Abb. 12).

### Oskar Scheuer (1876–1941)

Oskar Scheuer [102, 103] ist eher mit Publikationen zur Geschichte der Sexualität, des Sexualverhaltens oder zur Geschichte der Studentenschaft in Erinnerung geblieben, aber er veröffentlichte auch Arbeiten mit klinischem Bezug. Der Sohn eines jüdischen Altwarenhändlers studierte nach dem Besuch des Znaimer Gymnasiums ab 1896 an der Wiener Medizinischen Fakultät mit einem Semester Unterbrechung in Prag. Danach begann er 1903 als „Aspirant“ seine venerodermatologische Ausbildung am Rudolfstift in Wien bis zu seiner Niederlassung in Wien 1910. Er arbeite in den 1930er-Jahren eng mit dem Wiener Institut für Sexualmedizin von Leo Schidrowitz (1894–1956; [45, 46, 104]) im Hause des Amonesta-Verlags am Kohlmarkt 6 zusammen und war Autor mehrerer Lemmata im erfolgreichen „Handwörterbuch der Sexualwissenschaft“ [105] von Max Marcuse (1877–1963; [106]) oder im „Erotik Lexikon“ von Leo Schidrowitz [107]. Weiterhin war er an Vortragsveranstaltungen des Wiener Instituts für Sexualmedizin beteiligt [108].

**Abb. 13:**
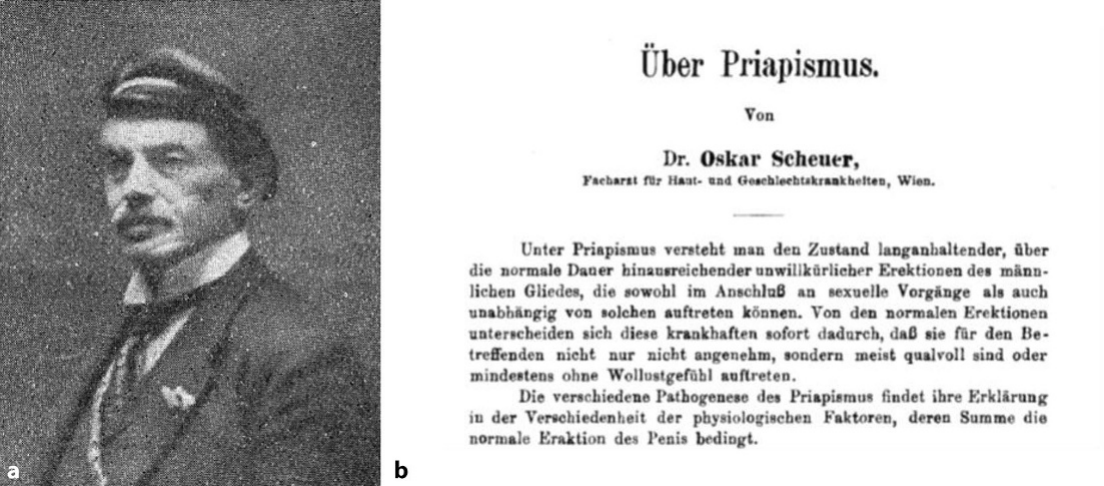
**a** Oskar Scheuer (1876–1941, Litzmannstadt/Łódź). (wikicommons). **b** Ausriss „Über Priapismus“ (Arch Derm Syphil [1909] 109, 449–496)

Während seiner Tätigkeit am Rudolfspital publizierte Oskar Scheuer einen um zwei eigene Fälle vermehrten Übersichtsartikel, in dem er sich der Diagnostik und Therapie des Priapismus zuwandte. Ferner veröffentlichte er mehrere Artikel zur Diagnostik und Behandlung von Geschlechtserkrankungen insbesondere der Syphilis [109] und der Gonorrhoe [110–112]. Auch seine Monographie zu den „Hautkrankheiten sexuellen Ursprunges bei Frauen“ von 1911 war diesem Schwerpunkt gewidmet [113]. Später konzentrierte er sich in Zusammenarbeit mit Leo Schidrowitz besonders auf kulturanthropologische Untersuchungen zur Sexualität ([114, 115]; Abb. 13).

### Viktor Blum (1877–1954)

**Abb. 14:**
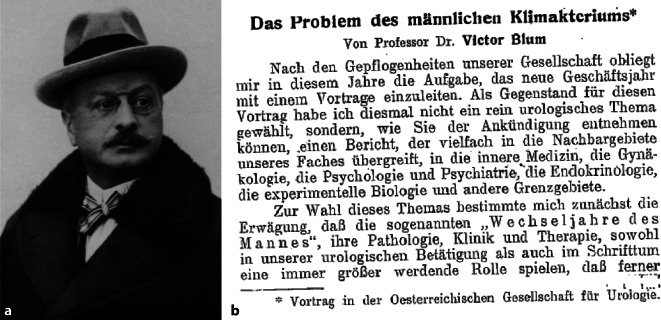
**a** Viktor Blum (1877–1954, Chicago). (Bildarchiv DGU, Repro Moll-Keyn, mit freundl. Genehmigung). **b** Ausriss „Das Problem des männlichen Klimakteriums“ (Wien Klin Wschr [1936], 44 Sp 1133–1138)

Der Wiener Urologe Viktor Blum (1877–1954) war ein wichtiger Repräsentant der Wiener Urologie in der Zwischenkriegszeit, bevor er von den Nationalsozialisten im Jahre 1938 nach dem „Anschluss“ Österreichs vertrieben wurde. Er studierte nach Besuch des Wiener Staatsgymnasiums (VIII. Bezirk) zwischen 1894–1900 Medizin ebenda, bevor er bis 1902 zunächst Assistent an der Allgemeinen Wiener Poliklinik in der Abteilung Chirurgie wurde, um dann bis 1908 in der Urologie bei Anton Ritter von Frisch (1849–1917), dem ersten Präsidenten der Deutschen Gesellschaft für Urologie, zu arbeiten. Im Jahre 1908 habilitierte sich Blum für „Urologie“ an der Wiener Medizinischen Fakultät, im Jahr 1921 wurde er zum a.o. Professor ernannt. Er war Primar am Wiener Sophienhospital. [116, 117]. Im Jahre 1908 verfasste er eine Monografie zur „Symptomatologie und Diagnostik der urogenitalen Erkrankungen“. Blum behandelt dort ausführlich die Themen Impotenz, krankhaften Samenverlust, Sterilität, den Symptomenkomplex der sexuellen Neurasthenie und die Masturbation. Blum räumte der Psychoanalyse und der Psychotherapie Freuds hier eine ergänzende Funktion für die Therapie ein und publizierte auch immer wieder Artikel im Grenzgebiet zwischen Urologie und Psychoanalyse ([118]; Abb. 14).

### Oswald Schwarz (1883–1949)

**Abb. 15:**
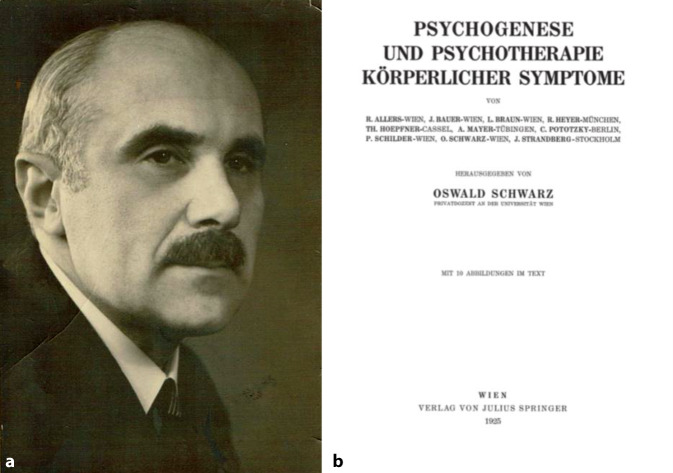
**a** Oswald Schwarz (1883–1949, London). (Institut für Geschichte der Medizin, Medizinische Universität Wien, mit freundl. Genehmigung). **b** Frontispiz Psychogenese und Psychotherapie körperlicher Symptome (Repro Moll-Keyn, mit freundl. Genehmigung)

Der Sohn eines Rechtsanwalts Oswald Schwarz [119, 120] war nach dem Medizinstudium in Wien ab dem Jahre 1912 als Arzt an der Urologischen Abteilung der Allgemeinen Wiener Poliklinik unter Anton Ritter von Frisch (1849–1917) tätig [121]. Im Jahre 1919 habilitierte er sich mit einer Arbeit über die „Störungen der Blasenfunktion nach Schußverletzungen des Rückenmarks“ an der Wiener Medizinischen Fakultät. Mit einer hierauf aufbauenden wegweisenden Zusammenstellung der Blasenphysiologie im Handbuch von v. Lichtenberg et al. positionierte er sich direkt im Differenzierungsbereich zwischen Urologie, Neurourologie und Sexualmedizin [122]. Nach dem Ersten Weltkrieg kam er in Kontakt mit Alfred Adler (1870–1937) und dessen Individualpsychologie, was ihn der Sexualmedizin und deren Forschungsansätzen noch näher brachte ([123–125]; Abb. 15).

Er wurde Dozent für Urologie an der Medizinischen Fakultät in Wien. Seine philosophisch-anthropologischen Vorträge brachten ihm seinen Spitznamen „Urosoph“ ein [122, 126]. Schwarz war zugleich operativ sehr ambitioniert, was die Akzeptanz seine sexualmedizinischen Arbeiten im Kreis der Mediziner wesentlich förderte [127]. Sogar seine Ausflüge auf das Terrain der Psychosomatik wurden von seinen Kollegen toleriert [128]. So konnte er interdisziplinäre Impulse für das Wissensfeld der Sexualmedizin beitragen ([129]; Abb. 15).

## Beiträge in Wiener Medizinischen Fachzeitschriften

**Abb. 16:**
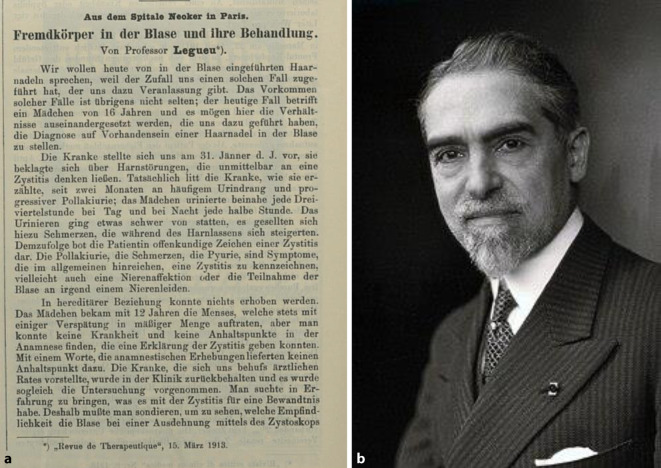
**a** Allgemeine Wiener Med. Zeitung 1913, **b** Paul Legeu (1863–1939) BIUM Paris, Repro Moll-Keyn, mit freundlicher Genehmigung

Zusätzlich zu den Arbeiten dieser in der Erinnerungskultur nicht stetig lebendig gebliebenen Autoren lassen sich in den in Wien erschienenen medizinischen Fachzeitschriften noch weitere Arbeiten finden, die den Grenzbereich zwischen Urologie und Sexualmedizin akzentuieren. Diese betonen oftmals die klinisch-praktischen Aspekte wie die sexualmedizinische Anamnese oder die geschickte klinische oder endoskopische Untersuchung. 1913 etwa wurde in der *Allgemeinen Wiener Medizinischen Zeitung* ein Artikel des renommierten Pariser Urologen Felix Legueu (1863–1939) nachgedruckt, der in zwei Ausgaben sehr differenziert das technische Vorgehen der Endoskopie bei Jugendlichen beschrieb ([130]; Abb. 16).

Bereits im Jahre 1903 war in der gleichen Zeitschrift ein umfangreicher Artikel über das einzige zu dieser Zeit für rational wirksam erachtete potenzsteigernde Mittel, Yohimbin, veröffentlicht worden. Es handelt sich um ein Pflanzen-Indol-Akaloid des Yohimbe-Baumes (C_21_H_26_N_2_O_3_, Cortex johimbe), das der Berliner Chemiker Leopold Spiegel (1865–1927) im Jahre 1900 beschrieben hatte, nachdem es die indigene Bevölkerung Zentralafrikas angeblich seit langem als Aphrodisiakum und Potenzmittel eingesetzt hatte. Yohimbin sollte einerseits den Geschlechtstrieb verstärken, andererseits gegen organisch verursachte Erektionsstörungen wirken. Bis zu diesem Zeitpunkt hatte es nur volksmedizinische Mittel wie Ginseng, Rhinozoroshorn oder Canthariden gegeben. Das C_21_H_26_N_2_O_3_ revolutionierte die Behandlung der erektilen Dysfunktion [131]. In den medizinischen Zeitschriften erschienen nach der pharmazeutisch-chemischen Darstellung und Patentierung nun immer wieder wissenschaftliche Artikel zum Wirkstoff, die dann im Anzeigenteil durch entsprechende Werbebotschaften untermauert wurden. Hersteller stritten darüber, wer ein reineres Extrakt darstellte. Bis zum Verkaufsstart der PDE-5-Hemmer im Jahre 1998 in Deutschland – der „zweiten sexuellen Revolution“ – war dieses Präparat die einzige rationale, im Doppelblindversuch belegte, wirksame Therapieoption für eine erektile Dysfunktion [132]. Neben Wiener Autoren waren es auch immer wieder renommierte Berliner Autoren in diesem Gebiete wie Albert Eulenburg (1840-1917), die hier dann das Präparat auch durch wissenschaftliche Artikel indirekt bewarben [133, 134]. In dem gleichen Heft dieser Wiener Zeitschrift findet man in Anschluss daran einen weiteren Fallbericht eines ausländischen Journals über eine penile Rekonstruktion [135].

Neben der pharmazeutischen Option wurden technische Hilfsmittel in der *Wiener Medizinischen Wochenschrift* vorgestellt. Der später in Auschwitz ermordete Militärarzt Otto Lederer (1872-1942 Ausschwitz-Birkenau) etwa publizierte hier eine Arbeit zu seiner Vakuum-Erektionshilfe, einem „Apparat zur künstlichen Erektion des Penis“ [136]. 1896 hatte der Civilingenieur Paul Gassen aus Köln eine Konstriktionsvorrichtung angegeben und diese in Zeitschriften wie u. a. dem Simplicissimus beworben, die Lederer wahrscheinlich unter Zuhilfenahme des Vakuums weiterentwickelte. Lederer verband die Wirkung eines gestuften Gummikompressionsringes mit der Volumenvergrößerung durch Vakuum. Vakuum war ab den 1830er-Jahren zur verschiedenen Therapiezwecken in der Medizin eingeführt und auch zur Behandlung der männlichen Impotenz eingesetzt worden [137, 138]. In den 1960er-Jahren wurde diese Idee durch Gedding D. Osbon wieder aufgenommen und von der FDA 1982 allgemein zur Therapie zugelassen [139].

## Fazit

Neben Berlin entwickelte sich Wien im „fin de siècle“ zur „Hauptstadt des Sex“ [140]. Einen Anteil daran hatten Mediziner wie urologisch tätige Chirurgen, Urologen, Venerodermatologen oder Psychiater, deren Arbeiten gleichzeitig ihrerseits vom Wiener Umfeld inspiriert wurden. Die hier vorgestellten Institutionen, Personen, ihre Arbeiten und der kleine Ausschnitt aus Beiträgen mit Bezug zu sexualwissenschaftlichen Themen aus Wiener medizinischen Fachzeitschriften unterstreichen, dass Problemen der Genitalsphäre in und um Wien eine breite Beachtung geschenkt wurde und wie Untersuchungstechniken, Therapieoptionen und sexualwissenschaftliches Wissen sich gegenseitig stimulierten. In einem Großstadtbereich, der sich in der „Wiener Moderne“ zu einem Schmelztiegel verschiedener Kulturen mit einer reichen Musik- und Literaturszene und einem innovativen Wissenschaftsraum entwickelt hatte, boten sich sexualwissenschaftlich interessierten Ärzten vielfache Möglichkeiten [141, 142]. Wichtige Protagonisten des neuen Fachgebiets, die in Wien studiert hatten und der Urologie nahe standen, erhielten dort ihre Fachprägung und bereicherten das Gebiet durch Behandlungsangebote für sexuell assoziierte Leiden und/oder Wünsche.

Einige Traditionslinien, die von den hier vorgestellten Personen in die heutige Urologie führen, waren lange unterbrochen, da viele von ihnen nach dem „Anschluss Österreichs“ 1938 fliehen mussten, nicht mehr wissenschaftlich tätig sein konnten, ermordet und aktiv aus dem Fachgedächtnis gestrichen worden waren. Der vorliegende Beitrag soll einerseits auch im Sinne einer Quellensammlung und -rekonstruktion diese Traditionslinien des Arbeitens im Grenzbereich zwischen Urologie und Sexualwissenschaft wieder offenlegen und dabei andererseits den Wert interdisziplinären Denkens unter Bezug auf die eigene Spezialität in Erinnerung rufen.

## Footnotes

1 Iwan Bloch (Berlin) wird in der Regel als derjenige bezeichnet, der den Begriff erstmals definitorisch benutzte und auch im Schrifttum hier auf die eigene Vorreiterrolle hinwies. Der Lebensreformer Karl Vanselow (1877–1959) verwendete ihn 1904 bei der Gründung seiner „Vereinigung für Sexualreform“, worauf V. Sigusch hinwies.

2 Angetter D, Hansson N 2020 Neun Mal nominiert – neun Mal gescheitert. Der Hormonforscher Eugen Steinach verpasste den Nobelpreis für Physiologie oder Medizin trotz mehrmaliger Nominierungen; online: https://www.billrothhaus.at/images/Angetter_Verhinderte-Nobelpreistraeger_Teil-1_Steinach.pdf. Zugegriffen 23.08.2023.

3 Bis zu einem Fachgebietsabkommen mit der Deutschen Gesellschaft für Chirurgie 1974 war es in Deutschland fast nicht möglich, einen Facharzt für Urologie ohne den gleichzeitigen Erwerb des Facharztes für Chirurgie zu erwerben, obwohl auf dem Deutschen Ärztekongress 1924 in Bremen beiden Fächern ein gleichrangiger Status zugesprochen wurde. Online: https://www.bdc.de/wp-content/uploads/2015/11/BDC-URO_1980.pdf Zugegriffen 15.08.2023.

4 Die Wiener Gesellschaft für Urologie wurde 1919 gegründet, sie bildete de facto den Nucleus für eine Gründung einer Österreichischen Gesellschaft für Urologie 1935 sowie wiederum nach dem 2. Weltkrieg im Januar 1947.

5 Archiv Univ Wien Senat Universität Wien S 305.92 Haslinger, Koloman, gestorben 09.03.1944, Nekrolog, k. A, Michael Hubenstorf 2012 The Nazi leader of urology in Vienna: Koloman Haslinger (1889–1944), in ders.: Urology and National Socialism in Austria, in: Dirk Schultheiss, Friedrich Moll: Urology under the Swastika, Davidsvonds uitgev. S. 29–32 online:.

6 Beiträge zu Albert Eulenburgs Real-Encyclopädie der gesamten Heilkunde. Erste Auflage, Band 2 (1880), S. 165–167: Beschneidung; S. 200–222 Band 3 (1880) Band 7 (1881) S. 44–50: Hypospadie Band 10 (1882) S. 402–422: Penis Band 11 (1882) S. 28–37: Präputium; S. 45–72: Band 14 (1883) S. 379–390: Varicocele.

7 Finger E 1888 (1. Aufl.), 1891 (2. Aufl.), 1893 (3. Aufl.), 1896 (4. Aufl.), 1901 (5. Aufl.), 1905 (6. Aufl.) Die Blenorrhoe der Sexualorgane und ihre Complicationen: Nach dem neuesten wissenschaftlichen Standpuncte und zahlreichen eigenen Studien und Untersuchungen dargestellt. Deuticke, Leipzig und Wien.
